# Strains and approaches for genetic crosses in the oleaginous yeast *Lipomyces starkeyi*

**DOI:** 10.1002/yea.3671

**Published:** 2021-10-15

**Authors:** Yuko Takayama

**Affiliations:** ^1^ Department of Biosciences, School of Science and Engineering Teikyo University Utsunomiya Tochigi Japan; ^2^ Division of Integrated Science and Engineering, Graduate School of Science and Engineering Teikyo University Graduate Schools Utsunomiya Tochigi Japan

**Keywords:** genetic crosses, heterothallic strain, *Lipomyces starkeyi*, oleaginous yeast, sporulation

## Abstract

The oleaginous yeast *Lipomyces starkeyi* is a powerful lipid producer with great industrial potential. Recent studies have reported the isolation of mutant *L. starkeyi* cells with higher lipid producing capacity. Although genetic engineering strategies have been applied to *L. starkeyi*, classical genetic approaches are lacking. The development of tools that facilitate genetic crosses in *L. starkeyi* would not only make it possible to build improved lipid‐producing strains but also facilitate molecular biological analysis of this species. In this study, I report a set of strains and approaches useful for performing genetic crosses with *L. starkeyi*. The homothallic *L. starkeyi* reportedly forms an ascus containing two to 20 spores. These spores were resistant to glusulase and could be dissected using a micromanipulator, suggesting that random spore and tetrad (spore dissection) analysis can be adapted for *L. starkeyi*. Additionally, to isolate a pair of heterothallic strains useful for genetic crosses, the homothallic strain was exposed to UV irradiation, and 10 self‐sterile strains were crossed with one another. One of these combinations, Ls75 and Ls100, sporulated stably. Moreover, to detect genetic recombination, I introduced a different drug resistance marker into each strain and crossed them. The resulting progeny exhibited Mendelian segregation of the resistance markers. Altogether, the work reported here provides a powerful resource for genetic analysis in *L. starkeyi*.

## Graphical abstract

The oleaginous yeast *L. starkeyi* forms multispores in an ascus. In this study, I report the isolation of crossable *L. starkeyi* strains (Ls75 and Ls100). The progeny of a cross between Ls75 and Ls100 exhibited Mendelian segregation. These crossable strains will be useful for genetic analysis and may provide insight into non‐conventional yeasts that form multispores.

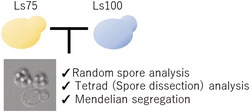

## 1 INTRODUCTION

*Lipomyces starkeyi* is a remarkable organism with regard to lipid production and has been extensively studied. In particular, it has been well studied for its capacity to produce and accumulate large amounts of lipids from many kinds of carbon sources and sewage sludge. In addition, the genome sequence of *L. starkeyi* has been determined (Riley et al., 2016), and some transformation methods have been reported (Calvey et al., 2014; Dai et al., 2017, 2019; Oguro et al., 2017; Takaku, Miyajima, et al., 2020). Using molecular genetic approaches, *L. starkeyi* recombinant strains have been identified, and the resulting strains are able to produce a variety of compounds (McNeil & Stuart, 2018; Takaku, Matsuzawa, et al., 2020). It would be valuable to further modify recombinant strains through genetic crosses; however, genetic cross strategies have not previously been reported for *L. starkeyi*.

In the established genetic yeasts *Saccharomyces cerevisiae* and *Schizosaccharomyces pombe*, sporulation can occur at high frequency (≧70%), and each ascus contains four spores (Briza et al., 1990; Plante & Labbe, 2019). The four spores in an ascus are the products of a single meiotic event, such that genetic analysis of the spores (‘tetrad analysis’) can reveal linkage relationships among genes. Tetrad analysis is also necessary for constructing strains useful for other genetic and biological studies. One reason similar methods have not been applied in *L. starkeyi* is that sporulation in this species is peculiar. Like other yeasts, *L. starkeyi* form asci; however, each ascus can contain from two to 20 spores (Kurtzman, 2011). Moreover, the frequency of sporulation of *L. starkeyi* is extremely low. Indeed, plating on commonly used sporulation media (V8, YM, or 1/10 YM) induces sporulation at a frequency of only 2%, and the frequency on *L. starkeyi*‐specific sporulation medium (AF medium) is 14.6% (Watanabe et al., 1997).

Another barrier to genetic analysis in *L. starkeyi* is that available strains behave as homothallic strains, whereas heterothallic strains are preferable for genetic strategies. In Saccharomycotina species, cell type is determined by the presence of *MATa* or *MATα* alleles at the mating type (*MAT*) locus. The stability of the mating type classifies yeast strains as members of one of two groups, heterothallic or homothallic. Heterothallic strains have a stable mating type and only mate with strains of different mating types, and therefore behave as self‐sterile. In contrast, homothallic strains can exist in either of two different forms, a primary and a secondary form (Hanson & Wolfe, 2017; Riley et al., 2016). Secondary homothallism by mating type switching has been well studied in *S. cerevisiae* and *Sz. pombe*. Additionally, methylotrophic yeasts, such as *Ogataea polymorpha*, *Ogataea minuta,* and *Komagataella phaffii* (Hanson et al., 2014; Maekawa & Kaneko, 2014; Yoko et al., 2019), contain one copy each of *MATa* and *MATα* loci, flanked by a pair of inverted repeat (IR) sequences. One of the *MAT* loci is proximal to the heterochromatic region of the genome (centromere or telomere), and its transcription is selectively repressed. Mating type is switched by invasion of the chromosomal region between the two *MAT* loci, swapping the positions of the active and repressed *MAT* loci. *L. starkeyi* is classified as a primary homothallic species. According to previous reports (Hanson & Wolfe, 2017; Krassowski et al., 2019; Riley et al., 2016), the *L. starkeyi* genome harbors two *MATa* genes (a1 and a2) and two *MATα* genes (α1 and α 2) but lacks DNA repeats near these genes, which are located close together on the same chromosome. The functions of the *MAT* genes and pheromone‐receptor interactions of *L. starkeyi* have not been elucidated.

*L. starkeyi* has the ability to form spores and its genome sequence suggests that it is homothallic (McNeil & Stuart, 2018; Riley et al., 2016). Heterothallic strains are able to mate with opposite mating types, thus allowing for genetic crossbreeding. However, heterothallic strains of *L. starkeyi* have not been reported. In this study, I isolated strains of *L. starkeyi* useful for genetic analysis. The strains Ls75 and Ls100 were isolated from cells that survived exposure to UV irradiation. When Ls75 and Ls100 were crossed, sporulation occurred practically and stably. Moreover, the progeny of a cross between these strains exhibited Mendelian segregation. The crossable strains of *L. starkeyi* newly generated in this work will be useful for genetic analysis and may provide insights into non‐conventional yeasts that form multi‐spores.

## 2 MATERIALS AND METHODS

### 2.1 Yeast strains and media

The yeast strains used in this study are shown in Table 1. Yeast strains were cultured in YPD medium (2% D‐glucose, 1% yeast extract, 2% polypeptone). Selection YPD media contained 100 μg/ml hygromycin B (Nacalai Tesque, 07296‐24) or 50 μg/ml zeocin (Thermo, R25001). Sporulation media used were as follows: AF medium (sucrose 2.85 g/L, aspartic acid 230 mg/L, glutamic acid 510 mg/L, KH_2_PO_4_ 245 mg/L, MgSO4·7H_2_O 660 mg/ml, FeCl_3_·6H_2_O 1.7 mg/L, MnSO_4_·6H_2_O 0.51 mg/L, ZnSO_4_·7H_2_O 4.5 mg/L, KOH 410 mg/ml, pH 6.5), YM medium (1% D‐glucose, 0.5% polypeptone, 0.3% yeast extract, 0.3% malt extract), 1/10 YM medium (YM medium diluted 10‐fold with distilled water). Solid media contained 2% agar (Sigma, A1296‐500G). Cells were incubated at 26°C for growth and crossing. Lipid‐inducing medium was as follows: S medium (5% D‐glucose, 0.5% (NH_4_)_2_SO_4_, 0.1% KH_2_PO_4_, 0.01% NaCl, 0.1% yeast extract, 0.05% MgSO_4_·7H_2_O, 0.01% CaCl_2_·2H_2_O) (Yamazaki et al., 2019).

**TABLE 1:** Strains used in this study

Strain Name	Genotype	Parental strain	Source or reference
*Lipomyces starkeyi* NBRC1289			JCM5995T CBS1807
*Saccharomyces cerevisiae* OC‐2			JCM1499
Ls75	*Ls75*	NBRC1289	This study
Ls100	*Ls100*	NBRC1289	This study
Ls75Z	*Ls 75 Δlslig4*::P_TDH3_‐*Sh ble*‐T_TDH3_	*Δlsku80 Δlslig4* x Ls75	This study
Ls100H	*Ls100 Δlsku80*::P_TDH3_ *‐hph‐*T_TDH3_	*Δlsku80 Δlslig4* x Ls100	This study
*Δlsku80 Δlslig4*	*Δlsku80*::P_TDH3_ *‐hph‐*T_TDH3_ *Δlslig4*::P_TDH3_‐*Sh ble*‐T_TDH3_		(Oguro et al., 2017)

### 2.2 Quantitation of sporulation

Cells grown on sporulation solid medium were picked up using sterile micropipette tips and were suspended in PBS (137 mM NaCl, 8.1 mM Na_2_HPO_4_, 1.47 mM KH_2_PO_4_, 2.68 mM KCl) and counted using an Olympus CX41. Sporulation frequency was calculated using the following equation: Sporulation frequency (%) = 100 × [asci/(asci + vegetative cells)]. The standard deviation from three independent experiments was calculated. At least 500 cells were counted.

### 2.3 Microscopy

Spore cells suspend in PBS were observed using a DM5500B digital microscope equipped with a 100× objective lens (N.A. 1.30) and a DFC 310FX digital CCD color camera (Leica Microsystems GmbH, Wetzlar, Germany).

### 2.4 Spore dissection

Sporulated cells were scraped and suspended in 20 μl of sterile water. The suspended cells were streaked on YPD plates. Spores on YPD plates were dissected using a micromanipulator (SporePlay+, Singer). Enzyme treatment (e.g., zymolyase) was not required, because the spores are released from the mature ascus (Kurtzman, 2011).

### 2.5 Random spore analysis

*L. starkeyi* cells were streaked on sporulation solid medium and incubated at 26°C. The sporulated cells were scraped and treated with 0.5% glusulase (Perkin Elmer, NE154) overnight at room temperature. The treated cells were collected by centrifugation (15,000×*g*, 3 min, 25°C) and washed with sterile water and suspended in 100 μl of sterile water. The cell suspension was vigorously mixed by a vortex mixer (Scientific Industries) for 1 min and sonicated in 500 μl of 0.01% Tween 20 solution. Cell suspensions (100 μl) were then plated on a YPD plates and incubated at 26°C for 5–7 days.

### 2.6 UV irradiation

The cells were spread on YPD plates, and the plates were exposed to a dose of 0 to 100 J/m^2^ of UV light (FUNA UV Crosslinker). After UV irradiation, the plates were incubated at 26°C. UV sensitivity was normalized by defining the number of colonies obtained without irradiation as 100%. Since the cell viability decreased when the UV dose was high, the numbers of spread cells were 300 and 3000 for 0 and 25 J/m^2^, and were 3000 and 30,000 cells for 75 and 100 J/m^2^, respectively.

### 2.7 BODIPY staining and microscopy analysis

S medium culture cells were collected by centrifugation (200×*g*, 3 min, 25°C) and then washed with PBS. The cell pellets were suspended in 25 μl of 0.5 μg/ml BODIPY™ 493/503 (Invitrogen, USA) and incubated for 10 min at room temperature. After washing with PBS, the cells suspended in PBS were observed using a TCS SP8 confocal microscope equipped with a 63× objective lens (N.A. 1.40) (Leica Microsystems GmbH).

## 3 RESULTS

### 3.1 Conditions that induce sporulation of *L. starkeyi*

Previous reports show that sporulation of *L. starkeyi* is induced on YM, 1/10 YM, and AF media (Kurtzman, 2011; Watanabe et al., 1997). The reported sporulation frequency of *L. starkeyi* CBS1807 is extremely low; YM and 1/10 YM induced sporulation at a rate of 2% and AF induced it at a rate of 14.6% (Watanabe et al., 1997). Disappointingly, there have been few reports on *L. starkeyi* sporulation. Identification of strains and/or conditions under which spore formation by *L. starkeyi* is stable would facilitate genetic studies. To accomplish this, I first confirmed whether the homothallic strain *L. starkeyi* NBRC1289 (also called CBS1807) could be induced to sporulate. *L. starkeyi* NBRC1289 cells grown on YPD plates were streaked on YM, 1/10 YM, or AF solid media and incubated at 26°C. After 1 month, sporulation was not observed on YM medium; however, I did observe a few sporulation events on 1/10 YM (3.0 ± 2.9%). The overall frequency of sporulation on AF medium was 17.3 ± 2.5% at 2 weeks after plating and did not increase much after further incubation, presumably as most spores had already been released by mature asci (Kurtzman, 2011). Consistent with a previous report (Watanabe et al., 1997), AF medium efficiently induced *L. starkeyi* sporulation. Thus, I primarily used this medium in subsequent experiments.

**FIGURE 1:**
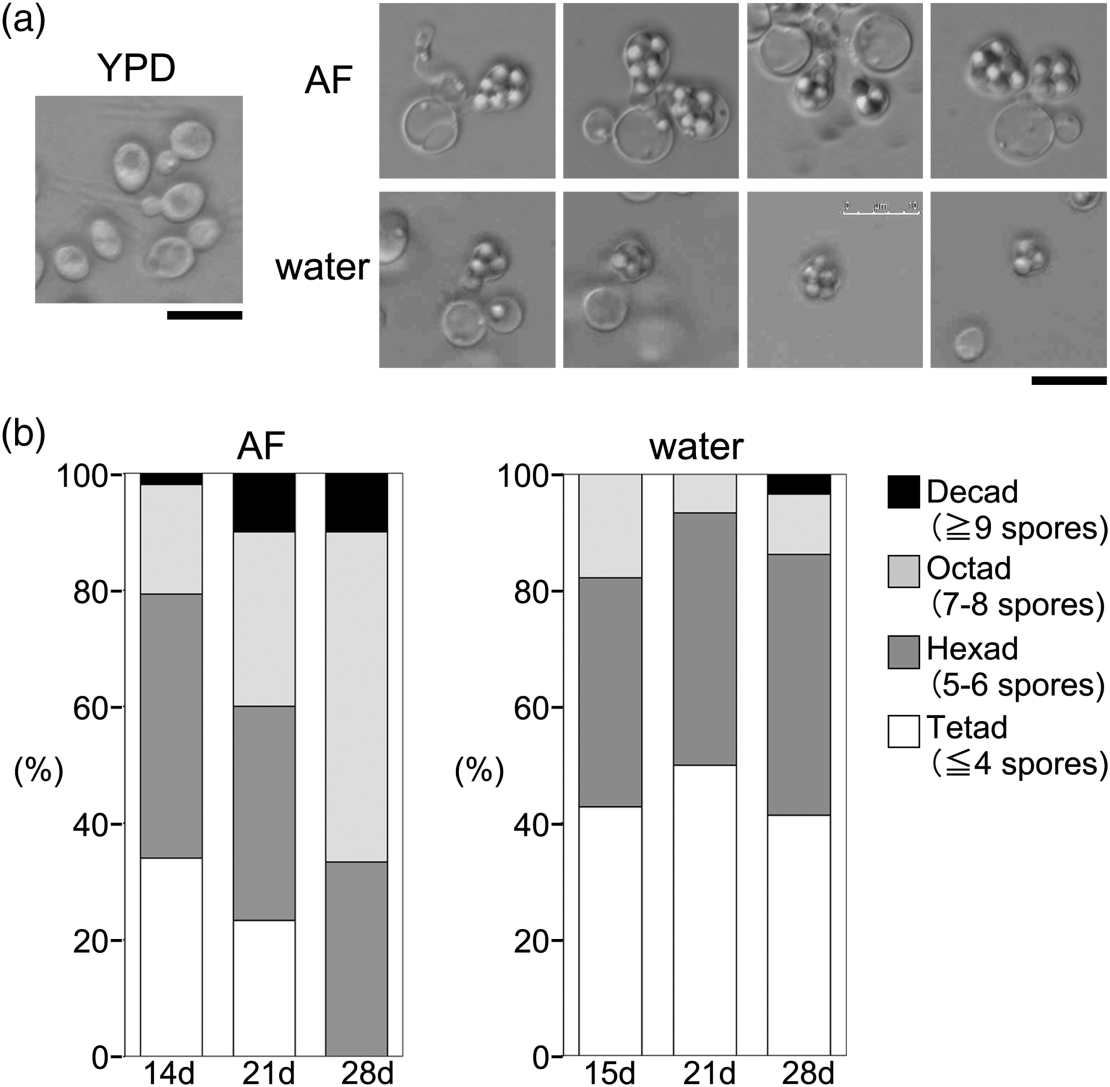
Sporulation of *L. starkeyi*. (a) Morphology of *L. starkeyi* asci. *L. starkeyi* NBRC1289 cells that sporulated on AF or water solid media were suspended in PBS and observed by microscopy. *L. starkeyi* NBRC1289 cells grown on YPD plates were also shown. Bar, 10 μm. (b) Quantification of spore number within asci. *L. starkeyi* cells were streaked on AF or water solid media. Spore numbers were counted for at least 50 asci (AF) or 30 asci (water)

Unlike the asci of other fungi, which produce four or eight spores, the *L. starkeyi* asci contain 2–20 spores (Figure 1a, upper panel) (Kurtzman, 2011). This multi‐spore appearance was the same as that observed for teliospores of *Rhodotorula* species (Newell & Hunter, 1970). To ascertain why there are variations in the number of *L. starkeyi* spores, I measured the number of spores every week for 1 month. After 14 days of incubation on AF solid medium, *L. starkeyi* predominantly formed hexads (i.e., asci with five to six spores). After 28 days, the strain predominantly formed octads (seven to eight spores), and the proportion of decads (≥9 spores) also increased (Figure 1b, left panel). Thus, the number of spores produced gradually increased with longer incubation times.

The number of spores in *Schizosaccharomyces octosporus* asci ranges from four to eight, and the number depends on nutrient conditions (Seike & Niki, 2017). With this in mind, I next ascertained whether the number of spores formed by *L. starkeyi* decreased when cells are streaked on media types with reduced nutrients as compared with AF medium. Surprisingly, *L. starkeyi* sporulation was induced on a plate containing only distilled water and 2% agar (Figure 1a, lower panel), albeit at a low frequency (6.0 ± 2.3%). In this ‘water solid’ medium, almost all asci formed tetrads or hexads, even after 28 days of incubation (Figure 1b, right panel), indicating that the number of spores produced was affected by nutrient conditions.

### 3.2 Spore analysis of *L. starkeyi*

**FIGURE 2:**
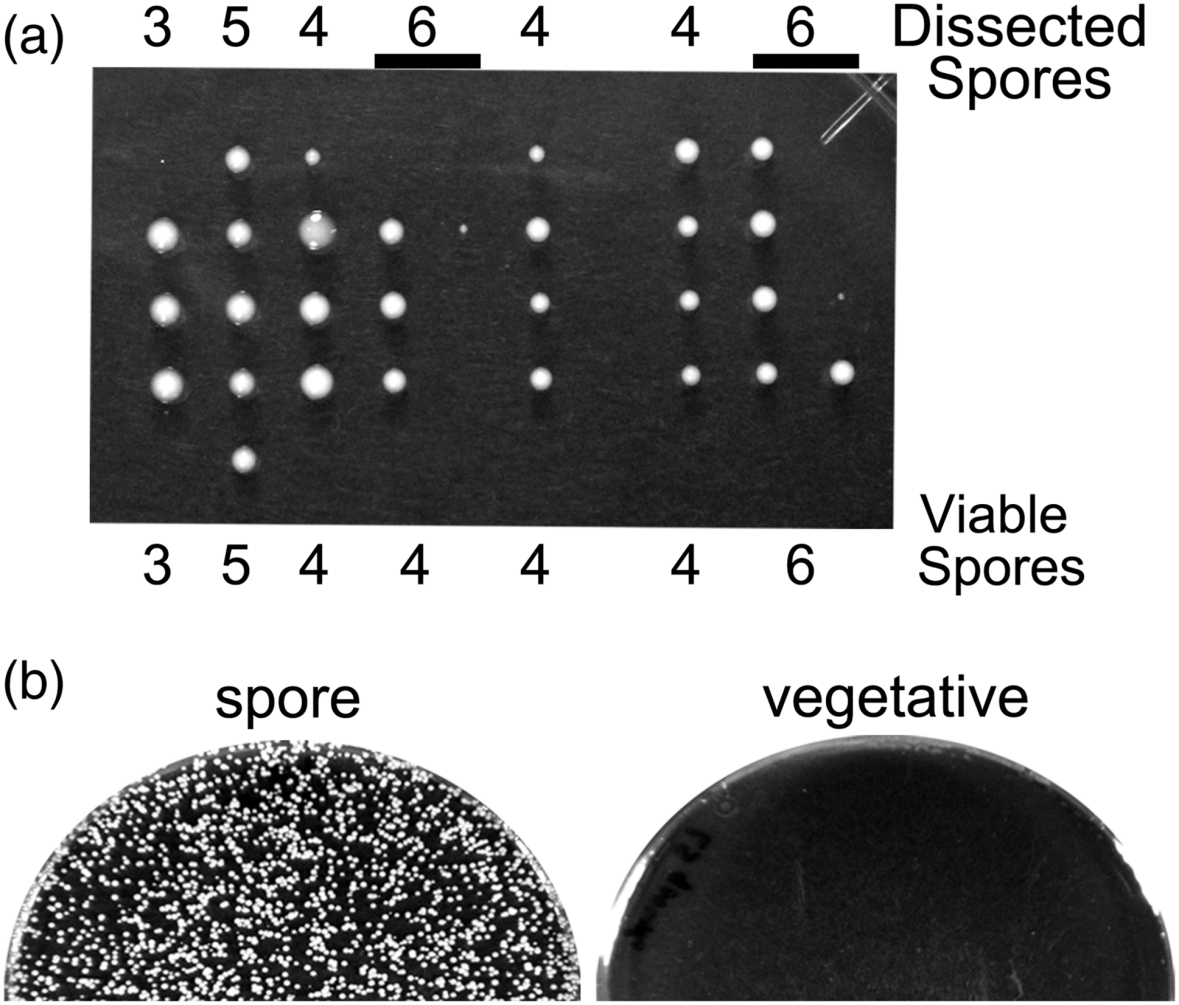
Analysis of *L. starkeyi* spores. (a) Dissection of *L. starkeyi* spores. The number of spores within an ascus (top) and the numbers of viable spores (bottom) are shown. (b) Glusulase treatment of spores. *L. starkeyi* NBRC1289 cells streaked on AF plates (spore) or YPD plates (vegetative) were treated with glusulase and spread on YPD plates

In yeast species, spore dissection (tetrad analysis) is useful not only for linkage studies, but also for building strains with multiple mutations. However, whether spores in the multi‐spore asci of *L. starkeyi* could be dissected was not known. To ascertain this, I induced *L. starkeyi* NBRC1289 cells to form spores on AF solid medium, then dissected ascospores onto YPD plates using a micromanipulator. I chose asci containing three to six spores as it was difficult to completely separate spores in asci with 10 or more spores. Most separated spores were viable (Figure 2a), and these segregants sporulated when cells were incubated on AF solid medium, indicating that the segregants maintained homothallism.

Spore dissection is a classical method for genetic analysis; however, this method requires use of a specialized instrument and is not appropriate when handling an ascus with many spores. By contrast, random spore analysis is a convenient and high‐throughput method. To be appropriate for random spore analysis, spore cells must be resistant to treatment with glusulase, an enzyme prepared from snail intestinal juices that can digest the ascus wall and kill vegetative cells. To test the suitability of this approach, I ascertained whether spores of *L. starkeyi* were resistant to glusulase. A 5‐mm diameter colony grown on AF solid medium was scraped and treated with glusulase. The treated cells were able to grow on YPD plates (Figure 2b, spore). In contrast, for a colony scraped from a YPD plate, glusulase‐treated cells did not grow (Figure 2b, vegetative). These results indicate that *L. starkeyi* spores are resistant to glusulase. Altogether, these results show that both spore dissection and random spore analysis can be performed with *L. starkeyi*.

### 3.3 Isolation of crossable strains

**FIGURE 3:**
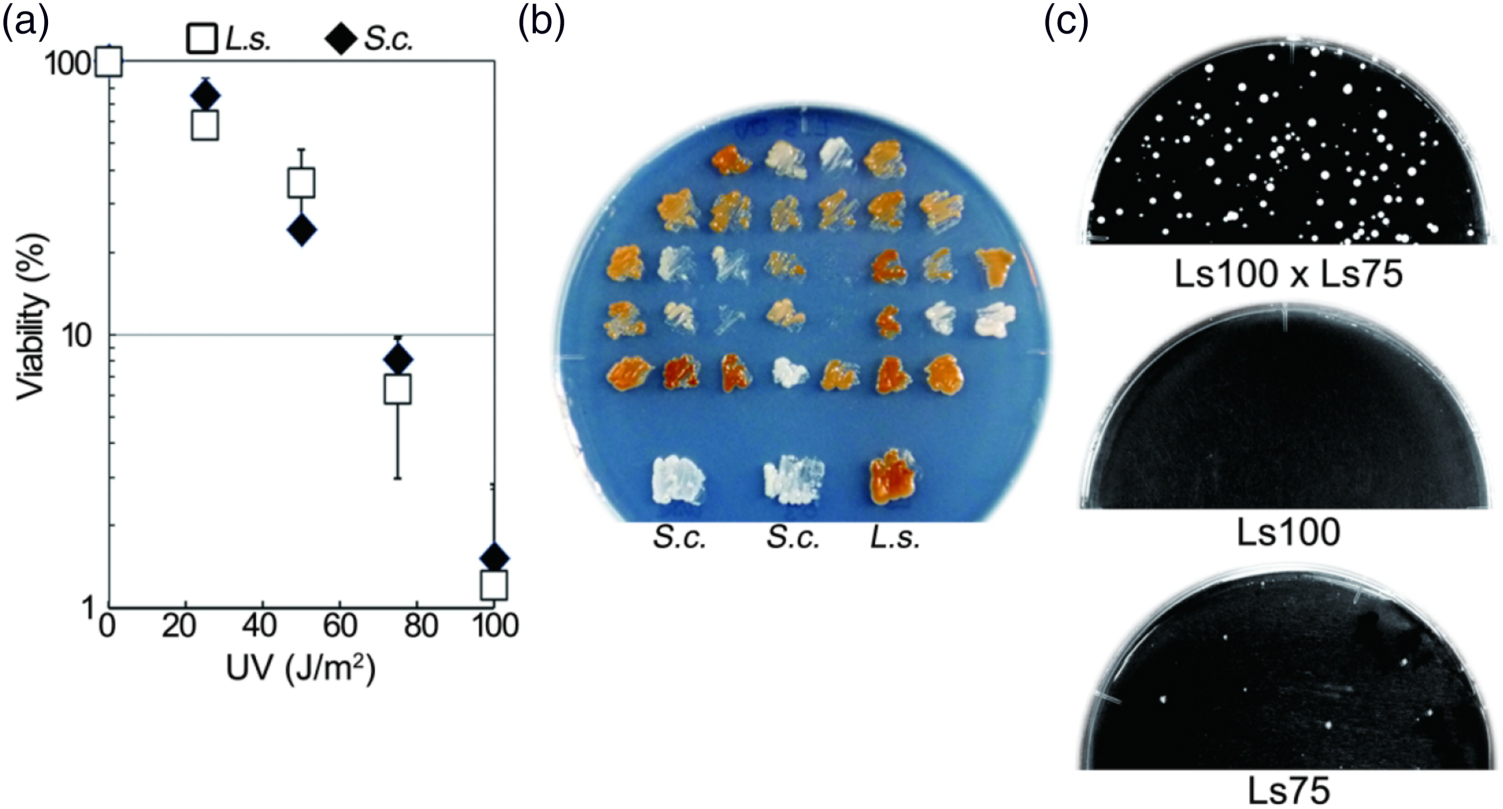
Isolation of genetically crossable strains of *L. starkeyi*. (a) UV sensitivity. *L. starkeyi* NBRC1289 cells (white boxes) and *S. cerevisiae* CO‐2 cells (black diamonds) were spread on YPD plates and exposed to UV. UV sensitivity was normalized by defining the number of colonies obtained without irradiation as 100%. The counts were performed independently three times. (b) Cells that survived exposure to UV were streaked on AF solid medium at 26°C. Well‐sporulated colonies were brown in color. Homothallic strains, *L. starkeyi* NBRC1289 (*L.s*.) and *S. cerevisiae* CO‐2 (*S.c*.), served as controls. (c) Random spore analysis. Cells from strain Ls100 alone and Ls75 alone or crossed with Ls100 and Ls75 on AF media were treated with glusulase and spread on YPD plates [Colour figure can be viewed at wileyonlinelibrary.com]

Heterothallic strains mate with cells of an opposite mating type. These strains are very useful for genetic studies with different genotypes. To obtain crossable strains, I decided to select self‐sterile cells following exposure to UV irradiation. To do this, I first determined the UV sensitivity of the *L. starkeyi* NBRC1289 strain. The cells were spread on YPD plates and exposed to UV irradiation. The UV sensitivity of *L. starkeyi* was the same as that for *S. cerevisiae* OC‐2 (Figure 3a). Lipid‐producing mutants have been isolated following optimal UV irradiation resulting in about 5% cell viability (Tapia et al., 2012). Therefore, I next irradiated cells with 75 or 100 J/m^2^, which resulted in a cell viability of 6.8 ± 1.8% or 1.2 ± 1.2%, respectively. The 111 survivor cells were then streaked on AF medium. The well‐sporulated *L. starkeyi* colonies changed to brown (Figure 3b). *S. cerevisiae* did not sporulate on AF medium. To select heterothallic cells unable to mate and sporulate with themselves, whitish colonies were picked up, and a lack of spore formation was confirmed by microscopy. Ten strains showed sterility or extremely low sporulation efficiency, and I made all possible crosses among these 10 strains. If heterothallic‐like strains were included, then some combinations would form spores. Consistent with this idea, some of the 10 strains were fertile when crossed with others. One of these combinations, Ls75 and Ls100, was able to stably induce sporulation on AF medium but not on water plates. I did not detect Ls75 spores alone by microscopy; however, glusulase‐resistant cells grew. Therefore, Ls75 alone did sporulate, albeit at a low frequency. By contrast, for Ls100, I did not detect the formation of glusulase‐resistant cells, suggesting that it never sporulates. Notably, however, when Ls75 and Ls100 were mixed, they reproducibly formed spores on AF medium (Figure 3c). These results suggest that Ls75 and Ls100 behave as heterothallic strains.

### 3.4 *L. starkeyi* crossable strains maintain lipid production capability

**FIGURE 4:**
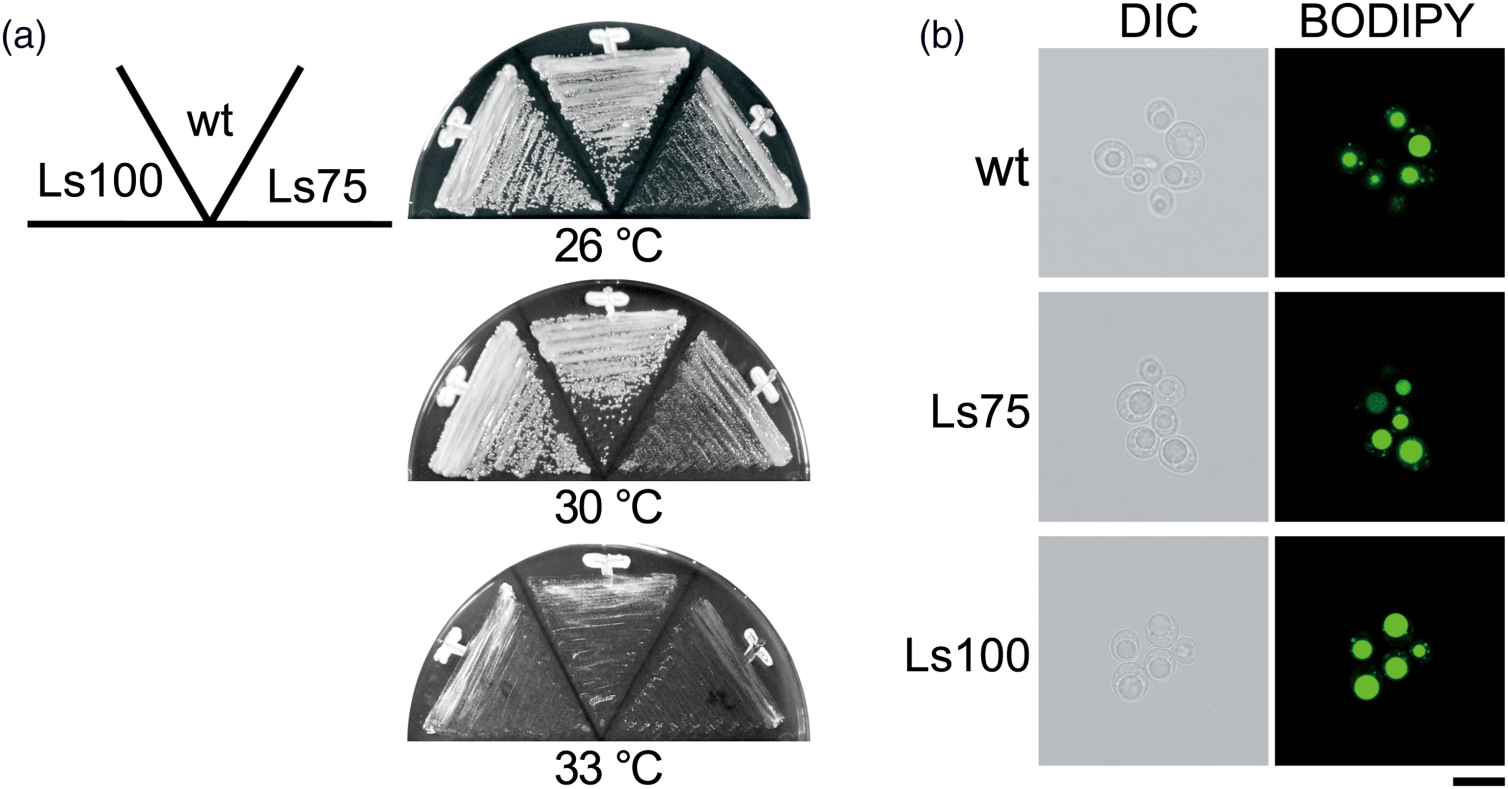
Phenotypes of crossable strains. (a) Growth phenotypes. *L. starkeyi* NBRC1289 (wt), Ls75, and Ls100 cells were streaked on YPD plates and incubated for 4 days at 26°C, 30°C, and 33°C. (b) Visualization of lipid droplets. *L. starkeyi* NBRC1289 (wt), Ls75, and Ls100 cells were cultured for 72 h in S medium and stained with BODIPY. Bar, 10 μm [Colour figure can be viewed at wileyonlinelibrary.com]

Ls75 and Ls100 strains were isolated after UV irradiation, which is known to induce mutations that may affect growth and lipid production. To define the growth phenotypes of these crossable strains, Ls75 and Ls100 and the original *L. starkeyi* cells were incubated on YPD plates at various temperatures. The three strains could grow at 30°C, but very little at 33°C (Figure 4a). No colony formed at 36°C (data not shown). The growth phenotype of Ls100 was identical to that of the original strain. However, Ls75 showed a slight growth delay compared with the original strain. Furthermore, the lipid productivity of these strains was tested. These strains were grown in S medium, which is a lipid induction medium (Yamazaki et al., 2019). Cells precultured in YPD medium were inoculated in S medium at a concentration of approximately 1 × 10^6^ cells/ml (0 h) and cultured for 72 h at 26°C. Lipid droplets of each cell were stained with BODIPY™ 493/503 and observed by microscopy (Figure 4b). There were no significant changes in lipid droplets in these cells. These results indicate that Ls75 and Ls100 strains display phenotypes comparable to the original *L. starkeyi* cells.

### 3.5 Genetic analysis of the *L. starkeyi* crossable strains

Because Ls100 and Ls75 behave as prototrophs, genetic recombination between these crosses could not be verified. To examine whether genetic recombination occurred during the crossing of these strains, Ls100 or Ls75 strains were crossed with the homothallic double‐mutant strain *Δlsku80::hph Δlslig4::Sh ble* (Oguro et al., 2017) in which the *hph* and *Sh ble* genes are inserted into the *LsKU80* and *LsLIG4* genes, conferring a hygromycin‐ and zeocin‐resistant phenotype. After treatment with glusulase, spores were grown on YPD plates containing hygromycin or zeocin to select for drug‐resistant progeny, which were subsequently streaked on AF medium to test for self‐sterility. After crossing Ls100 with the hygromycin‐ and zeocin‐resistant strain, I identified a strain ‘Ls100H’ that was resistant to hygromycin but not to zeocin. This strain was self‐sterile but could be crossed with Ls75, resulting in sporulation. Likewise, a self‐sterile zeocin‐resistant strain, Ls75Z, was obtained after crossing the double‐mutant strain with Ls75. Ls75Z could be crossed with Ls100, similar to the parental strain Ls75. Notably, the phenotypes of these heterothallic drug‐resistant strains, Ls100H and Ls75Z, were different from either of the parental phenotypes, suggesting that meiotic recombination occurred when the homothallic *Δlsku80::hph Δlslig4::Sh ble* strain was crossed with the heterothallic Ls100 or Ls75.

**FIGURE 5:**
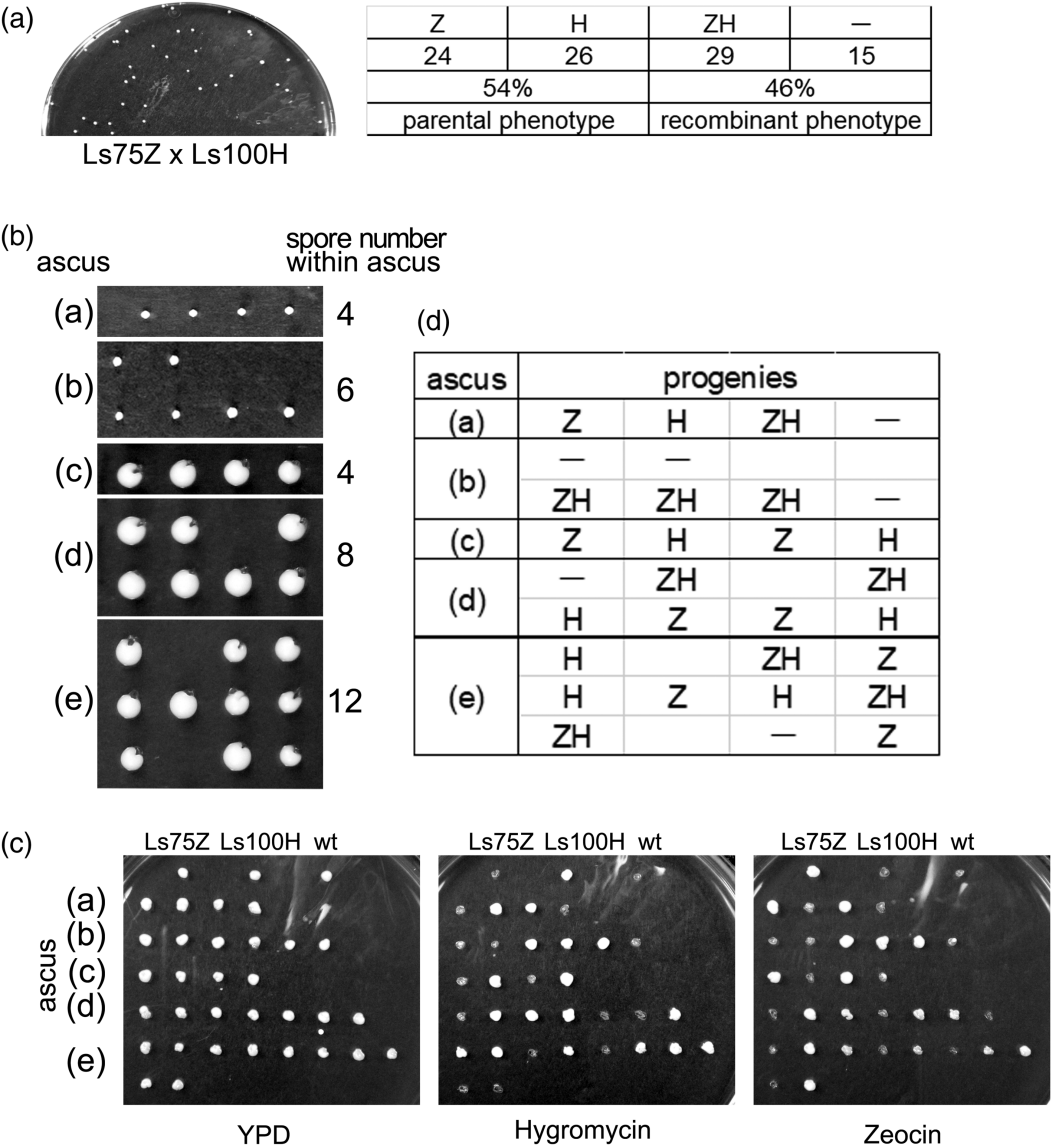
Genetic analysis of *L. starkeyi*. (a) Random spore analysis following a cross between Ls75Z and Ls100H. Cells resulting from a cross between Ls75Z and Ls100H were treated with glusulase and spread on a YPD plate (left). A total of 94 colonies were tested for hygromycin or zeocin resistance. Zeocin resistance and hygromycin sensitive cells (Z), zeocin sensitive and hygromycin resistance cells (H), zeocin and hygromycin resistance cells (ZH), and drug sensitive cells (−). (b) Dissection of spores resulting from a cross between Ls75Z and Ls100H. The number of spores in each ascus is shown. (c) The progenies shown in (b) were spotted on YPD, hygromycin, or zeocin plates and incubated at 26°C for 3 days. Ls75Z, Ls100H, and *L. starkeyi* NBRC1289 (wt) cells were spotted at the top as controls. (d) The phenotypes of the progeny in (c) are shown

Next, spores from a cross between Ls75Z and Ls100H were treated with glusulase, and I evaluated viable colonies for drug resistance phenotypes (Figure 5a). The proportion of cells exhibiting the parental phenotype (i.e., resistant to either zeocin or hygromycin) was 54%, and the proportion exhibiting a recombinant phenotype (i.e., resistant to both drugs or sensitive to both) was 46%. The genome sequence of *L. starkeyi* has been determined (Riley et al., 2016), but all contigs have not yet been joined. Thus, the physical distance between the markers (*LsKU80* and *LsLIG4*) was not clearly defined.

Lastly, to determine if genetic recombination analysis by spore dissection is possible, ascospores resulting from a cross between Ls75Z and Ls100H were dissected using a micromanipulator (Figure 5b). The progeny of five asci were tested for drug sensitivity (Figure 5c,d). Since the drug resistance genes are inserted in two different genes in the parental cells, a Mendelian inheritance pattern should result in progeny that exhibit a parental ditype (PD), a non‐parental ditype (NPD), and a tetra type (T). The progeny of ascus (a) had phenotypes consistent with two parental and two recombinant genotypes (i.e., T). The progeny of ascus (b) all had phenotypes consistent with a recombinant genotype (NPD). The progeny of ascus (c) all had phenotypes consistent with parental genotypes (PD). In addition, in asci (d) and (e), not all progeny were viable. Ascus (d) contained eight spores, one of which was inviable. Of the seven viable progeny, four showed the parental phenotype and three showed a recombinant phenotype. Ascus (e) contained 12 spores, two of which were inviable. Of the 10 viable progeny, six had parental phenotypes and four had recombinant phenotypes. I infer that the inviable spores were of a recombinant type that is sensitive to both drugs. These findings clearly demonstrate that genetic recombination occurred and was accurately detected following a cross of strains Ls75Z and Ls100H. Taken together, these results show that genetic analysis can be performed by crossing the *L. starkeyi* derivate strains Ls75 and Ls100.

## 4 DISCUSSION

*L. starkeyi* is a remarkable organism with the ability to accumulate lipids at a level that comprises up to 70% of its dry weight using various available carbon sources (McNeil & Stuart, 2018; Takaku, Matsuzawa, et al., 2020). Despite *L. starkeyi* having excellent characteristics for industrial use, classical genetic cross strategies have not previously been reported. The identification of genetically crossable strains of *L. starkeyi* would facilitate the combining of mutations, such as mutations that have a positive impact on lipid production. In this report, I show that *L. starkeyi,* which forms multi‐spores, can be adapted for genetic analysis, and that the strains Ls75 and Ls100 together provide a powerful resource for genetic analysis.

*L. starkeyi* asci contain two to 20 spores, and the number of spores depends on incubation time and nutrient conditions (Figure 1). This multi‐spore appearance is the same as for the teliospores of *Rhodotorula* species (Newell & Hunter, 1970); however, the process of multi‐spore formation has not been elucidated. A previous report showed that when over‐replication at premeiotic S‐phase was induced by ectopic activation of cyclin B/cyclin‐dependent kinase, the asci contained up to 20 spores. Moreover, the formation of an ascus containing more than four nuclei occurred immediately following the execution of meiosis II and reached a maximum shortly thereafter (Strich et al., 2004). *L. starkeyi* predominantly formed hexads (five to six spores) at 14 days and octads (seven to eight spores) at 28 days (Figure 1b, left panel). Thus, the number of spores gradually increased with longer incubation times. If the multi‐spore asci of *L. starkeyi* form via over‐replication at the premeiotic S‐phase, then meiotic division and spore formation would be more rapid. In a study of *Sz. octosporus*, the number of spores was found to be dependent on nutrient conditions, and eight spores were not always formed (Seike & Niki, 2017). The authors mention that if eight spores are formed within an ascus in some fission yeast species due to an extra post‐meiotic mitosis, the regulation of spore number probably occurs at the level of commitment to post‐meiotic mitosis. Consequently, I propose that the multi‐spore asci of *L. starkeyi* might be the result of repeated rounds of post‐meiotic mitosis.

I found that an *L. starkeyi* homothallic strain can form spores not only on AF medium but also on water solid medium (Figure 1). Little is known about how water solid medium induces the sporulation of *L. starkeyi*. It has been reported that sporulation of the sake yeast Kyokai No. 7 is promoted by the presence of trace amounts of divalent cations present in agar powder (Suizu, 1996). If sporulation is promoted by divalent cations, sporulation frequency should be increased by the addition divalent cations. However, I found that adding magnesium or calcium ions or both to water solid medium did not increase the sporulation frequency and that the frequency was not affected when I used agar powder from another supplier (Wako) (data not shown). In addition, on water solid medium, I never observed sporulation after mixing Ls75 and Ls100. The sporulation frequency of the cross between Ls75 and Ls100 was lower than that of the homothallic strain (Figures 2b and 3c). Even homothallic *L. starkeyi* have a lower sporulation frequency on water solid medium (6.0 ± 2.3%) than on AF solid medium (17.3 ± 2.5%). Thus, it is possible that Ls75 and Ls100 can form spores on water solid medium, but the rate is so exceptionally low that these events were not detected.

This study shows that together, Ls75 and Ls100 provide a powerful resource for genetic analysis of *L. starkeyi*. Because the sites of mutation in these strains have not been identified, it is unclear if Ls75 and/or Ls100 have mutations at the *MAT* locus. Some of the progeny of a cross between Ls75 and Ls100 have a homothallic phenotype, suggesting incomplete linkage between these mutation sites. The *L. starkeyi* genome harbors *MATa* (a1 and a2 genes) and *MATα* (α1 and α 2 genes) loci that are close to one another (~20 kb) on the same chromosome (Hanson & Wolfe, 2017; Krassowski et al., 2019; Riley et al., 2016). The *MAT* loci of methylotrophic yeasts, such as *Ogataea* species and *K. phaffii* (Hanson et al., 2014; Maekawa & Kaneko, 2014; Yoko et al., 2019), are similar to that of *L. starkeyi*, and mating‐type switching has been identified. The mating type of these yeast is changed by swapping the positions of the active and repressed *MAT* loci, a process that requires the IR sequence (Maekawa & Kaneko, 2014). However, IR sequences have not been found in *L. starkeyi*. The mechanisms by which the mating type can be switched in the absence of an IR have not yet been revealed (Hanson & Wolfe, 2017). The authors of a previous study pointed out that mating type switching, which requires a mechanism to repress non‐expressed copies of the *MAT* genes, evolved in the Saccharomyces lineage relatively soon after the methylation of heterochromatin at the enhanced histone H3 at lysine 9 (H3K9) was lost (Riley et al., 2016). *L. starkeyi* is the only Saccharomycotina species that contains orthologs of genes involved in the formation of heterochromatin via H3K9 methylation. In particular, I have reported that the *L. starkeyi* heterochromatin protein Lsw1 can associate with heterochromatin in *Sz. pombe* in an H3K9 methylation‐dependent manner, suggesting that the expression of *MAT* locus genes might be repressed by heterochromatin via methylation of H3K9 (Takayama, 2020). Thus, analysis of the mechanisms of mating type switching using the newly isolated crossable strains, Ls75 and Ls100, might reveal a novel *MAT* determination system that is not dependent upon an IR sequence.

## CONFLICT OF INTEREST

The author declares no conflicts of interest.

## Data Availability

The data that support the findings of this study are available from the corresponding author upon reasonable request.
